# A luminescent Nanoluc-GFP fusion protein enables readout of cellular pH in photosynthetic organisms

**DOI:** 10.1074/jbc.RA120.016847

**Published:** 2020-12-04

**Authors:** Shungo Nakamura, Nae Fu, Kumiko Kondo, Ken-Ichi Wakabayashi, Toru Hisabori, Kazunori Sugiura

**Affiliations:** 1 Laboratory for Chemistry and Life Science, Institute of Innovative Research, Tokyo Institute of Technology, Midori-ku, Yokohama, Japan; 2 School of Life Science and Technology, Tokyo Institute of Technology, Midori-ku, Yokohama, Japan

**Keywords:** pH indicator, BRET, thylakoid membrane, cyanobacteria, plastoquinone pool, BRET, bioluminescent resonance energy transfer, COX, cytochrome c oxidase, DBMIB, 2,5-dibromo-3-methyl-6-isopropyl-p-benzoquinone, DCMU, 3-(3,4-dichlorophenyl)-1,1-dimethylurea, EYFP, enhanced yellow fluorescent protein, FR, far-red, PC, plastocyanin, PQ, plastoquinone, Rluc, Renilla luciferase

## Abstract

pH is one of the most critical physiological parameters determining vital cellular activities, such as photosynthetic performance. Fluorescent sensor proteins capable of measuring *in situ* pH in animal cells have been reported. However, these proteins require an excitation laser for pH measurement that may affect photosynthetic performance and induce autofluorescence from chlorophyll. As a result, it is not possible to measure the intracellular or intraorganelle pH changes in plants. To overcome this problem, we developed a luminescent pH sensor by fusing the luminescent protein Nanoluc to a uniquely designed pH-sensitive GFP variant protein. In this system, an excitation laser is unnecessary because the fused GFP variant reports on the luminescent signal by bioluminescence resonance energy transfer from Nanoluc. The ratio of two luminescent peaks from the sensor protein was approximately linear with respect to pH in the range of 7.0 to 8.5. We designated this sensor protein as “luminescent pH indicator protein” (Luphin). We applied Luphin to the *in situ* pH measurement of a photosynthetic organism under fluctuating light conditions, allowing us to successfully observe the cytosolic pH changes associated with photosynthetic electron transfer in the cyanobacterium *Synechocystis* sp. PCC 6803. Detailed analyses of the mechanisms of the observed estimated pH changes in the cytosol in this alga suggested that the photosynthetic electron transfer is suppressed by the reduced plastoquinone pool under light conditions. These results indicate that Luphin may serve as a helpful tool to further illuminate pH-dependent processes throughout the photosynthetic organisms.

Since all enzymatic reactions are strongly affected by the pH of the reaction conditions, pH is a critical factor for most physiological phenomena in living cells. In chloroplasts, the pH of the lumenal side of the thylakoid membrane and that of the stroma are important factors in determining photosynthetic performance. For example, in addition to pH as a determinant of ATP synthase catalyzed reaction rate, the pH difference (ΔpH) across the thylakoid membrane determines the ATP synthesis rate because ΔpH is used as the driving force for ATP synthesis (1, 2). Moreover, the enzymatic reaction steps regarding Calvin–Benson–Bassham cycle are also strongly influenced by pH in the chloroplast stroma (3, 4, 5). Despite the importance of these pH factors in photosynthetic reactions, *in situ* pH changes inside the chloroplast have never been determined. The photosynthetic reactions under the fluctuating light conditions are well studied in the present decades by mainly using pulse-amplitude modulation chlorophyll fluorescence measurement. The strong impacts of short-period fluctuation of the light intensity for photosynthesis performance are then described very well. Nevertheless, pH changes under these conditions cannot be determined because of the lack of the appropriate method to measure the intracellular or intraorganelle pH.

Gene-encoded pH-sensitive fluorescent proteins are one of the possible solutions for this challenge. For use in plant cells, there are two major requirements. The first is that their working pH range should include the expected pH changes. The second is that the signal colors should not overlap with the absorption ranges of pigments, such as chlorophyll, xanthophyll, and carotenoids in chloroplasts. To date, several fluorescent-type pH sensor proteins have been reported, such as pHluorin (6), pHusion (7), and GFpH (8). However, there are two major problems in using them for pH measurement in photosynthetic organisms. First, the excitation light for measurement may promote light-dependent reactions in chloroplasts. These probes are of course inapplicable under dark conditions. Second, their pKa values are too low to observe pH changes in chloroplasts. The expected pH values in chloroplast stroma are reported to be between pH 7.0 (dark) and 8.0 (light) (9, 10, 11, 12).

To overcome the problems in applying the current pH sensor proteins in the chloroplast, we have developed a novel luminescent-type pH sensor protein to measure the intracellular pH of photosynthetic organisms using the bioluminescent resonance energy transfer (BRET) mechanism. In 1999, Xu *et al.* (13) reported the first application of BRET by using *Renilla* luciferase (Rluc) (14) and enhanced yellow fluorescent protein (EYFP) (15) and succeeded in measuring the interaction of KaiB protein *in vitro* and *in vivo*. After this work, Hoshino *et al.* (16) reported that Rluc–EYFP fusion protein (BAF-Y) exhibited higher BRET efficiency and brighter emission and succeeded in single-cell bioluminescence imaging in 2007. Finally, Suzuki *et al.* (17) reported NanoLuc–Venus fusion protein (YeNL). NanoLuc (Nluc) (18) is the brightest luciferase derived from the *Oplophorus* luciferase. Venus is an EYFP derivative, which is 30-fold brighter than EYFP (19). YeNL was then applied to their Ca^2+^ indicator and successfully monitored *in vivo* Ca^2+^ dynamics (17).

Because the light intensity of the luminescent proteins is weak enough and does not influence the proton gradient formation across thylakoid membranes coupled with photosynthetic electron transfer reactions, the luminescent protein was thought to be a good tool for the GFP-based pH sensor proteins. Accordingly, we fused pH-sensitive GFP variant protein and NanoLuc (Nluc) and obtained the desired pH sensor protein Luphin (luminescent pH indicator protein) that enabled the measurement of the linear signal changes from pH 7 to 8.5. Although it is still difficult to measure pH changes in the thylakoid lumen, we have successfully used this sensor protein to observe unexpected pH changes in the cytoplasm of cyanobacteria during the light–dark transition.

## Results

### Development of a BRET-based pH sensor protein

**Figure 1:**
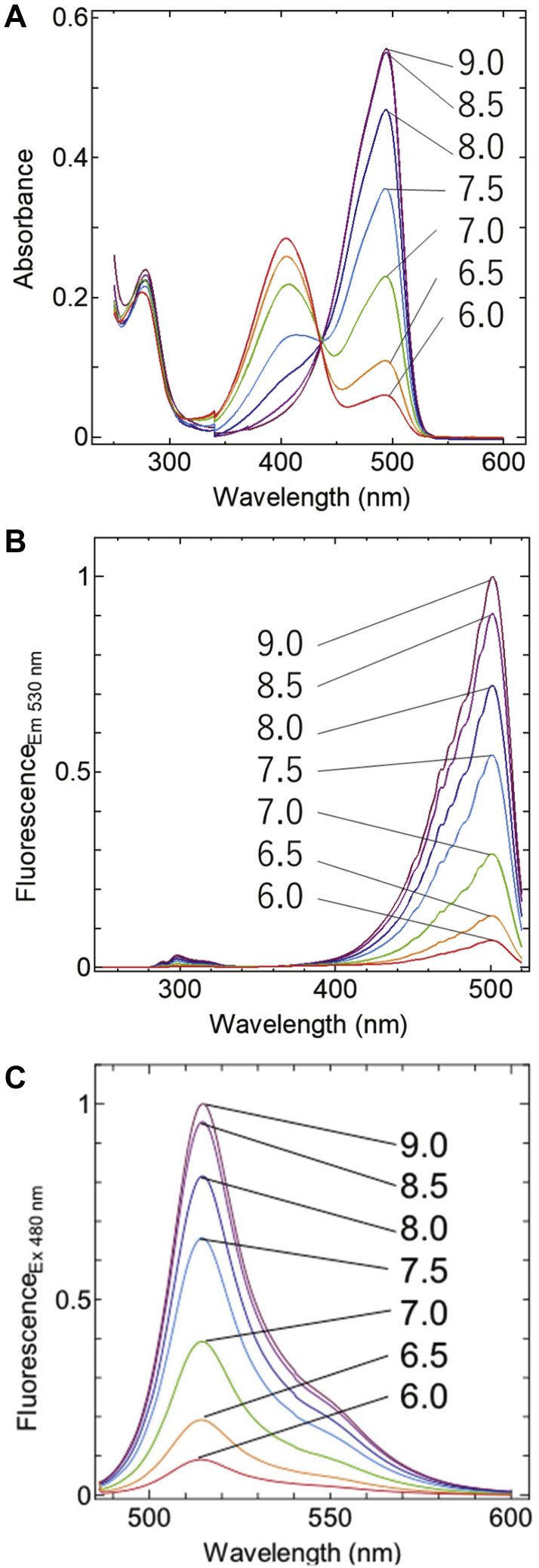
**The spectroscopic property of EGFP**_**pH**_**.***A*, the absorption spectra of EGFP_pH_ in buffers with various pH values. *B*, the fluorescence excitation spectra at 530 nm emission in buffers with various pH values. *C*, the fluorescence emission spectra at 480 nm excitation in buffers with various pH values.

First, we developed a pH-sensitive GFP variant by mutating the Enhanced GFP (EGFP). EGFP is a GFP variant that emits 509 nm fluorescence by 395 nm and 488 nm excitation light, and its pKa is 6.0 (20). We introduced three types of mutations. The first one contains T203V and S205V mutations that delete the excited-state intramolecular proton transfer (ESIPT) pathway (21). ESIPT happens when a protonated (neutral) chromophore is excited by a 395 nm light, and electron transfer occurs immediately so that the chromophore becomes anionic before emitting fluorescence. This process is called ESIPT, and because of this phenomenon, the emission wavelength from the protonated chromophore is maintained at the same wavelength of 509 nm as anionic chromophores. Therefore, the abovementioned mutations may result in the loss of 509 nm fluorescence for the 395 nm excitation light. Because protonated chromophores are dominant at lower pH conditions, the 509 nm emission caused by BRET will be weak enough at lower pH, when the GFP variant is fused with Nluc. However, the pKa of the chromophore is expected to increase due to T203 V mutation, because it is known that removing T203 increases the pKa (22). Next, 147iG and H148S mutations were introduced, which are close to the hydroxy group of the chromophore and are expected to affect the hydrogen bond interaction and adjust pKa to be at approximately 7.5. Mutations at the same position (insertion just behind 147th amino acid and substitution at 148th amino acid) were already applied and the folding of GFP was confirmed (23, 24). The third mutation is a part of the superfolder mutations (25) that stabilize the protein structure. In a series of superfolder mutations, S30R, Y39N, F99S, N105T, V163A, and I171V were adopted, because the other mutation positions are located close to the chromophore or the mutation points included in the first and second group, and may affect the ionization process of the chromophore. Eventually, the EGFP variant displayed a fluorescence spectrum with an emission peak at 513 to 515 nm when excited at 480 nm. In the pH range of 7.0 to 8.5, the fluorescence intensity increased with increasing the ambient pH (Fig. 1). Here, we named this variant EGFP_pH_.

**Figure 2:**
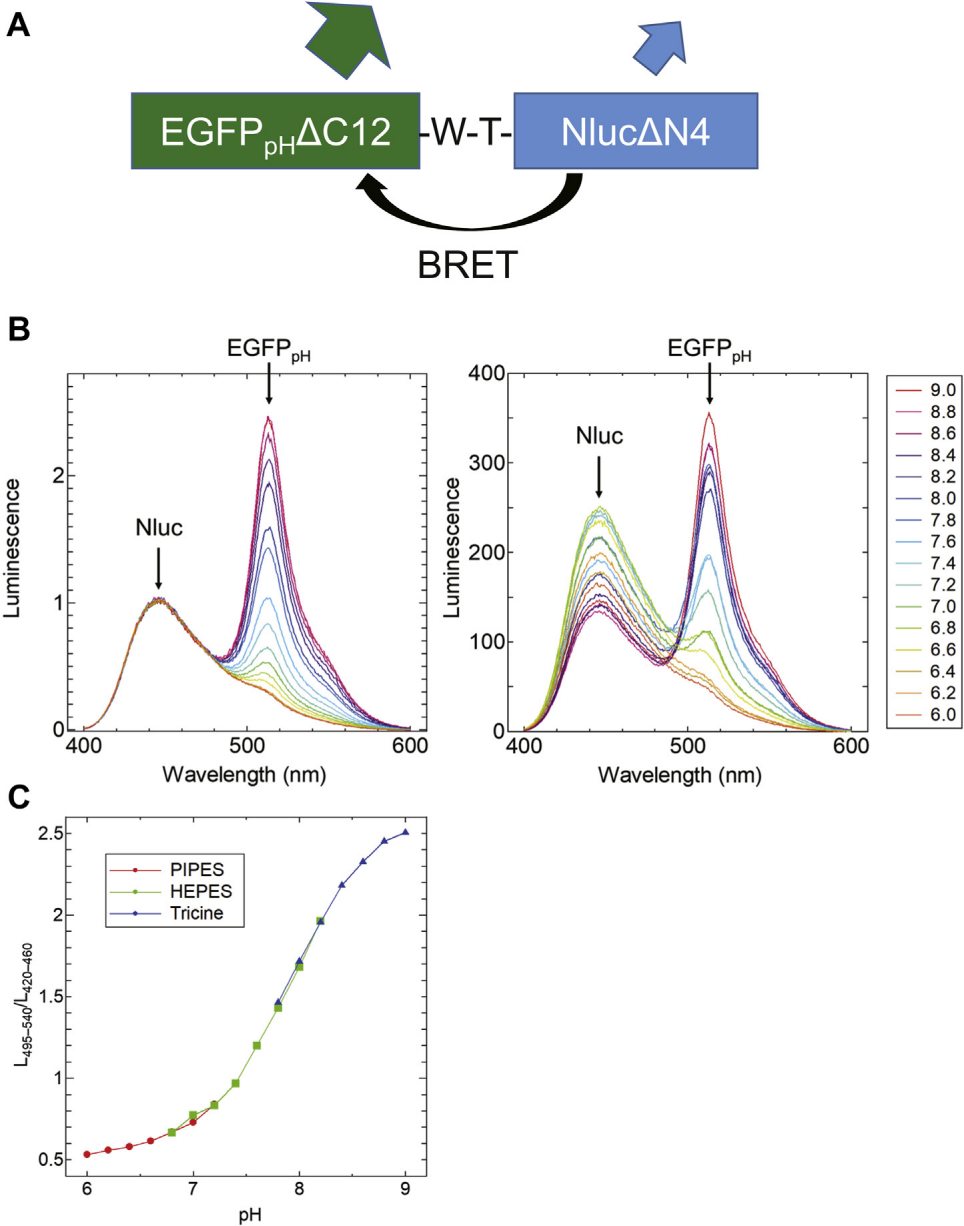
**The pH response of Luphin.***A*, the protein construct of Luphin. *B*, the normalized (left) and nonnormalized (right) luminescence spectra of Luphin in buffers with various pH values. In the left panel, the luminescence intensity was normalized to the peak at 450 nm. The emission peaks at 450 nm and 513 nm were derived from Nluc and EGFP_pH_, respectively. *C*, the pH response of Luphin calculated by L_495–540_/L_420–460_. L_495–540_ and L_420–460_ were the sum of the luminescence signal intensities in the 495 to 540 nm and 420 to 460 nm range measured at 1 nm intervals, respectively.

Second, we fused Nanoluc (Nluc) with EGFP_pH_. Nluc is a bioluminescent protein that emits 450 nm luminescence by adding the substrate “Furimazine” (18) and can be utilized as an efficient donor of BRET (17). The conjugated protein consists of EGFP_pH_ΔC12 (EGFP_pH_ lacking C-terminal 12 amino acids), the linker amino acid sequence of Trp-Thr (W-T), and NlucΔN4 (Nluc lacking N-terminal 4 amino acids) (Fig. 2*A*) and displayed the pH-dependent change of luminescent spectra (Fig. 2*B*). The absorption spectra under various pH values of the conjugated protein are shown in Fig. S1. As shown in Figure 2*C*, the luminescent signal ratio from EGFP_pH_ to the luminescence intensity of Nluc nearly shows the linear relationship against pH in the range of 7.0 to 8.5, which is the pH range expected within the chloroplast stroma as previously described (9, 10, 11, 12). We, therefore, named this sensor protein Luphin (luminescent pH indicator protein).

### Stability of the BRET signal from Luphin

**Figure 3:**
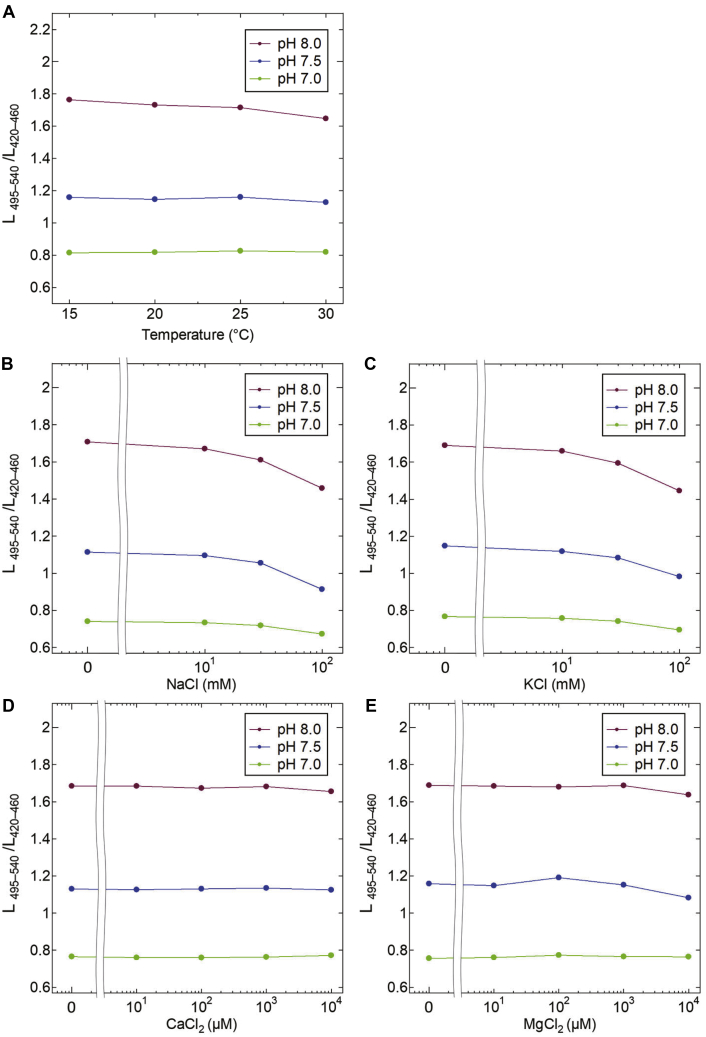
**The physiological stability of the luminescence signal of Luphin.***A*, luminescence signals in buffers with pH 7.0, 7.5, and 8.0 at different temperatures were measured. *B*–*E*, luminescence signals in buffers with pH 7.0, 7.5, and 8.0 were measured in the presence of NaCl (*B*), KCl (*C*), CaCl_2_ (*D*), and MgCl_2_ (*E*).

To confirm the stability of the BRET signal from Luphin, we examined the performance of Luphin under varying temperatures at different pH levels. As shown in Figure 3*A*, the signals were mostly stable, whereas a decrease in the BRET ratio signal was observed at 30 °C. Next, we examined the influence of cations (Na^+^, K^+^, Ca^2+^, and Mg^2+^) on Luphin signals. The luminescence ratio was stable in the presence of 0 to 30 mM Na^+^ and K^+^ (Fig. 3, *B*–*C*) and 0 to 1 mM Ca^2+^ and Mg^2+^(Fig. 3, *C*–*D*). These results indicate that Luphin is suitable for measuring pH changes in living cells.

### pH changes under the photosynthetic conditions

**Figure 4:**
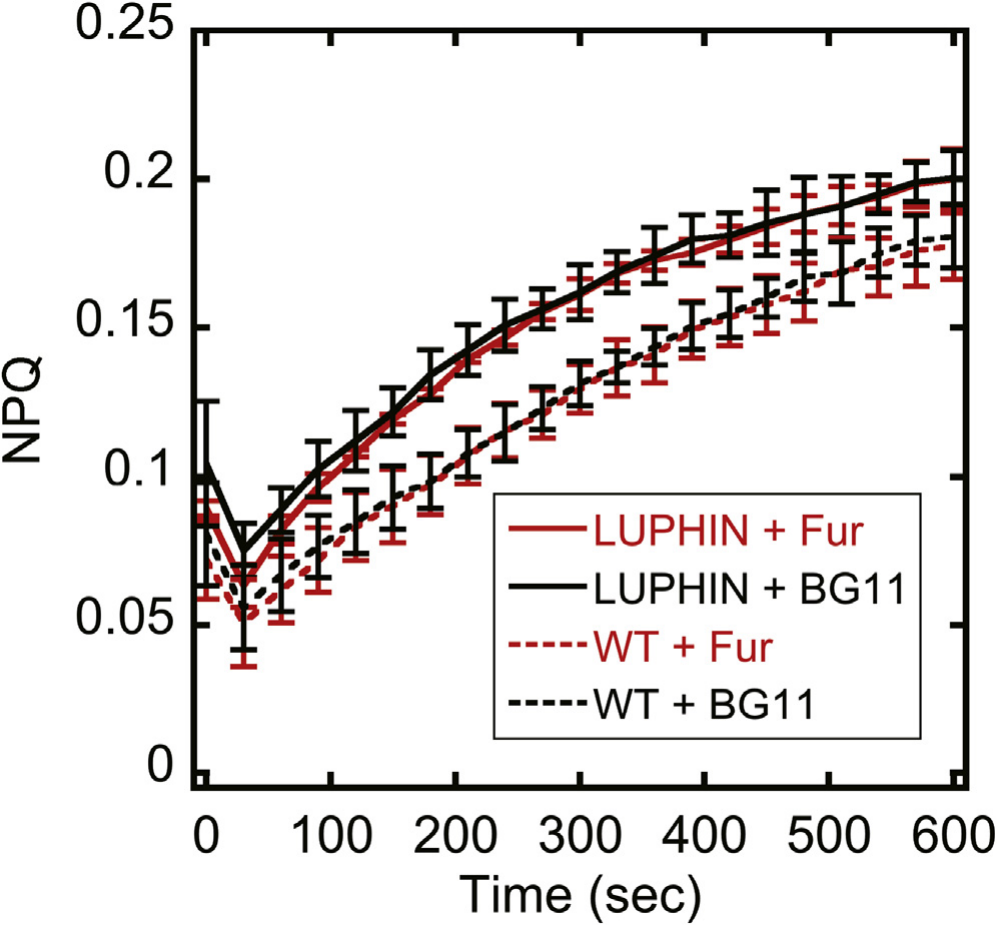
**The photosynthetic activities of Luphin-expressing *Synechocystis* measured by chlorophyll fluorescence.** Nonphotochemical chlorophyll fluorescence quenching (NPQ) under illumination of red actinic light (40 μmol m^−2^ s^−1^) of a Luphin-expressing (LUPHIN) and the wild-type (WT) *Synechocystis* cells was measured using DUAL-PAM 100 Chlorophyll Fluorometer (Walz). Prior to the measurements, cells were dark-acclimated for 10 min, followed by mixing with Furimazine solution (+Fur) or BG-11 medium (+BG11), and incubation in the dark for another 10 min. X-axis indicates the time (s) after actinic light exposure. Data are the mean ± SD from three independent experiments. Fv/Fm and ΦII were shown in Table S1.

To monitor the cytosolic pH changes under photosynthetic conditions, Luphin was expressed in the cytosol of the cyanobacteria *Synechocystis* sp. PCC 6803 (*Synechocystis*), one of the model phototrophs in the photosynthesis studies. The expression of the sensor protein in the cell was confirmed by using the western blotting method (Fig. S2). Before the pH change measurement, we have measured the PAM signal from the cyanobacteria containing Luphin and compared them with those from the WT cells to confirm that the luminescence from Luphin does not affect the photosynthetic parameters such as NPQ and ΦII (Fig. 4, Table S1).

**Figure 5:**
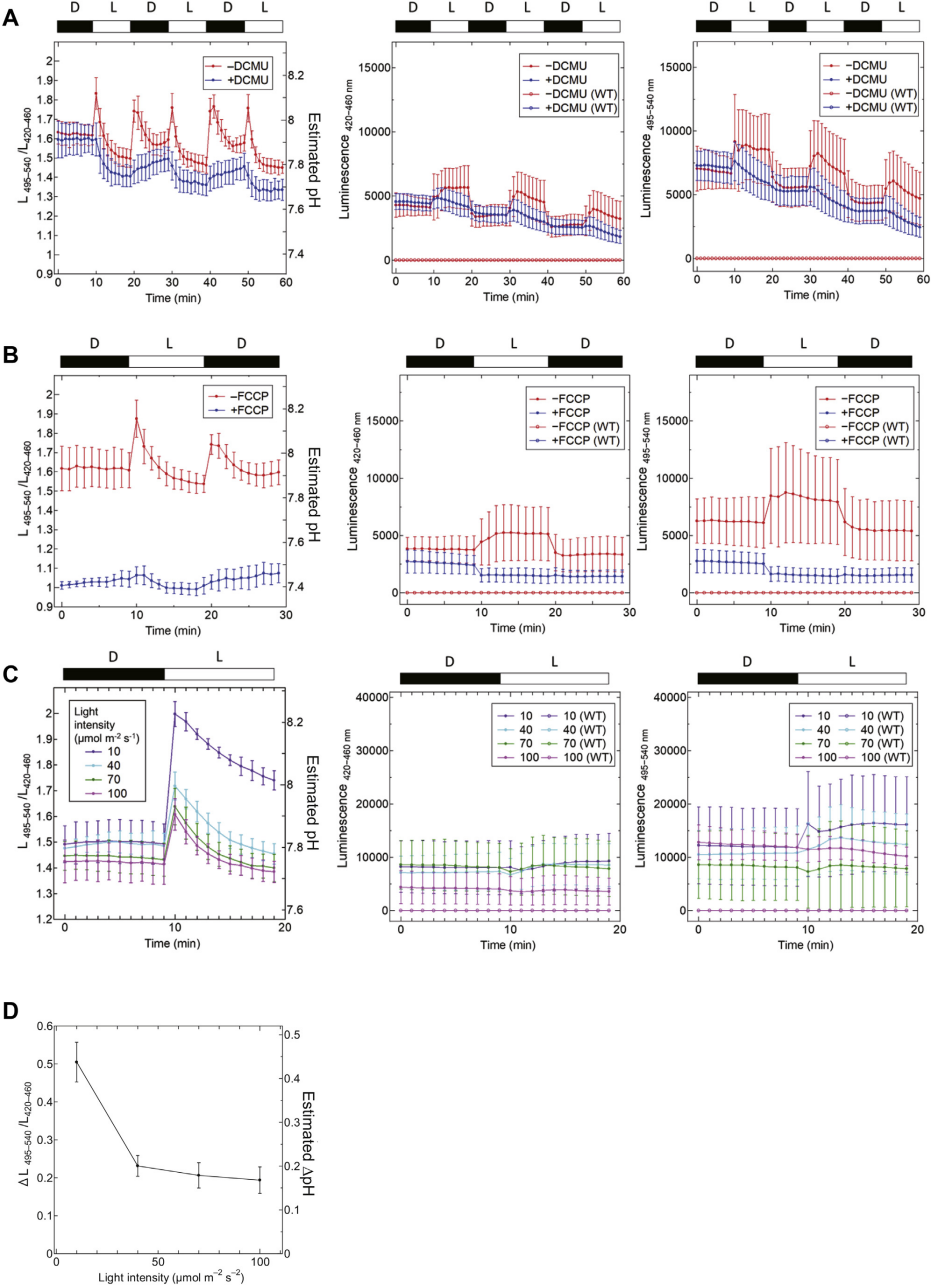
**The *in vivo* pH measurement of *Synechocystis* cytosol.***A*, the L_495–540_ (right panel) and L_420–460_ (center panel) values in the presence (*blue*) or absence (*red*) of 10 μM DCMU (*blue*) were measured, and the L_495–540_/L_420–460_ values (left panel) were calculated. Dark condition (D: 0 μmol photons m^−2^ s^−1^) and white light condition (L: 40 μmol photons m^−2^ s^−1^) were exchanged every 10 min. *B*, the L_495–540_ (right panel) and L_420–460_ (center panel) values in the presence (*blue*) or absence (*red*) of 30 μM FCCP were measured, and the L_495–540_/L_420–460_ values (left panel) were calculated. The light conditions are same as those for *A*. *C*, the L_495–540_ (right panel) and L_420–460_ (center panel) values in the dark and after light irradiation of 10, 40, 70, and 100 μmol photons m^−2^ s^−1^ were measured. The L_495–540_/L_420–460_ values (left panel) were then calculated. The open symbols indicate the luminescence intensities from WT cyanobacteria that did not express Luphin. All the signals were measured in the presence of furimazine. Because the luminescent signals from the WT cells were too weak, the blue and red lines overlapped. *D*. The L_495–540_/L_420–460_ values and the estimated pH within 1 min after light irradiation of 10, 40, 70, and 100 μmol photons m^−2^ s^−1^ were measured (n = 9).

Then, the Luphin signals in *Synechocystis* cells were measured under light (white light of 40 μmol photons m^−2^ s^−1^) and dark conditions (Fig. 5*A*). The signals obtained from the original *Synechocystis* cells (WT) were shown as controls (Fig. 5, open symbols). When the cells were incubated in the dark, the Luphin signals showed that the estimated cytosolic pH was approximately 7.9. Although the pH immediately increased up to 8.0 just after turning on the light, the rapid pH decrease was observed even in the light conditions, and it reached 7.8 within 10 min. When the light was turned off, the intracellular pH unexpectedly increased to 8.0, and then, it was not maintained and finally decreased to 7.8. The estimated pH increase caused by light or dark transition was mostly suppressed (Fig. 5*A*, blue line) when the cells were incubated with 10 μM 3-(3,4-dichlorophenyl)-1,1-dimethylurea (DCMU), a photosynthetic electron transfer pathway inhibitor, suggesting that the observed changes of estimated pH were caused by photosynthetic electron transfer activity.

To confirm that the observed signals shown in Figure 5*A* were caused by the proton transfer across thylakoid membranes, the uncoupling chemical carbonyl cyanide-p-trifluoromethoxy phenylhydrazone (FCCP) was applied. The intracellular pH in the dark became 7.4 by the addition of 30 μM FCCP to the culture medium, which was at the same level as the BG11 medium used for *Synechocystis* culture (Fig. 5*B*, blue line). As expected, the Luphin signal did not respond to the light/dark transition in the presence of FCCP.

Next, the light intensities of 10, 40, 70, and 100 μmol photons m^−2^ s^−1^ were used to investigate the light-intensity-dependent pH changes. The estimated pH increase within 1 min after irradiating dark-adapted *Synechocystis* was measured. As shown in Figure 5, *C*–*D*, the estimated pH increases after irradiation became small with increasing light intensity in the range of 10 to 100 μmol photons m^−2^ s^−1^. We could not examine the lower irradiance that was less than 10 μmol photons m^−2^ s^−1^ due to the limit of the irradiation facility in the laboratory.

### Investigation of the mechanisms of the pH changes

**Figure 6:**
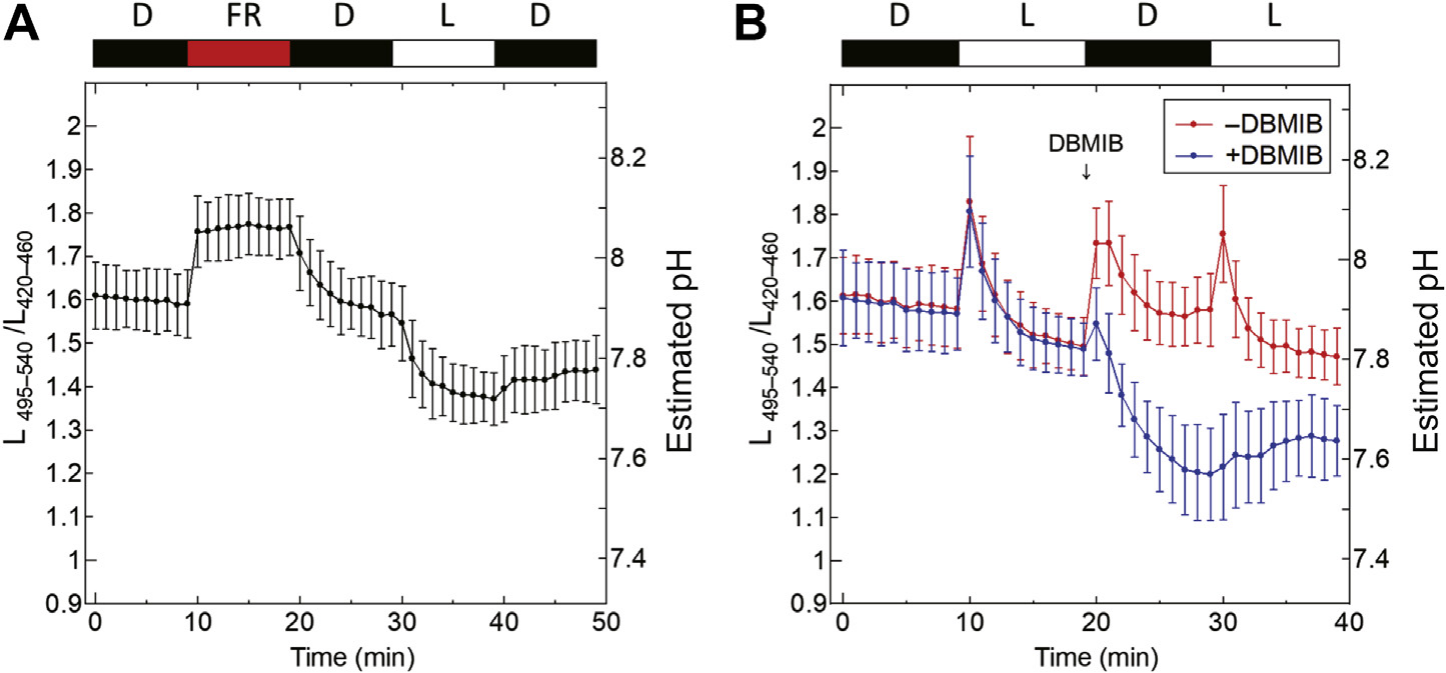
**The relevance between pH change and the inhibition of electron transfer system.***A*, the L_495–540_/L_420–460_ values were measured in the presence of 10 μM DCMU. *Synechocystis* was irradiated by FR light (730 nm peak, 40 μmol photons m^−2^ s^−1^) instead of white light from 10 min to 19 min. *B*, the effects of DBMIB on the L_495–540_/L_420–460_ values were measured and the estimated pH was calculated. In total, 10 μM DBMIB was added at 19 min (*blue symbols*) (n = 9).

The gradual decrease of the estimated pH during light irradiation and the increase just after turning the light off were unpredicted, although we successfully observed pH changes associated with photosynthetic electron transfer activities. To elucidate the cause of the gradual pH decrease during light irradiation, we examined the effect of far-red (FR) light (40 μmol photons m^−2^ s^−1^ with peak emission at 730 nm) in the presence of 10 μM DCMU (Fig. 6*A*). Only the cyclic electron transfer pathway will work under these conditions because FR light only excites PSI, and DCMU inhibits plastoquinone (PQ) reduction at the PSII site. The PQ pool may be therefore rather oxidized than the situation with the white light irradiation. Interestingly, the increase of the estimated pH caused by turning on the FR light was maintained during the FR light irradiation for 10 min (Fig. 6*A*); however, it was then decreased in the dark. The next white light irradiation in the presence of DCMU did not induce the pH shift (Fig. 6*A*) as indicated in Figure 5*A*. These results suggest that the gradual pH decrease during white light irradiation may have been induced by PQ pool reduction, and ATP synthase must first dissipate the formed proton gradient across thylakoid membranes to synthesize ATP. In contrast, the estimated pH increase observed after turning off the light was mostly suppressed by the addition of 10 μM 2,5-dibromo-3-methyl-6-isopropyl-p-benzoquinone (DBMIB) (Fig. 6*B*). This result suggests that the estimated pH increase after turning off the light is related to the oxidation of plastoquinol (PQH_2_) since DBMIB inhibits PQH_2_ oxidation at the cytochrome *b*_*6*_*f* (Cyt *b*_*6*_*f*) site, which is accumulated during irradiation.

## Discussion

### Advantages of Luphin

In this study, we developed a novel luminescent-type pH sensor protein “Luphin.” By adding the substrate “Furimazine,” Luphin emitted two luminescence peaks at 450 nm and 513 nm. The ambient pH can be measured as a ratio of these two luminescence intensities as shown in Figure 2*B*. Unlike other fluorescent-type sensors, Luphin does not require excitation light for measurement, because it is a BRET sensor that fuses pH-sensitive GFP with Nluc. This working mechanism has significant advantages when applied to photosynthetic organisms because photosystems are hardly excited by luminescence from Nluc, and autofluorescence from chlorophyll molecules is hardly induced during measurements. It is also suitable for use in weakly alkaline spaces, such as chloroplast stroma and cyanobacterial cytoplasm, as the emission ratio varies dynamically and linearly over a pH range of 7.0 to 8.5.

For measuring pH in plant cells, Luphin has several advantages over the previously reported BRET-type pH sensor, pHlash (26). In this sensor protein, *Renilla* luciferase “Rluc8” (27) was fused with a YFP variant “cpVenus” (28). The Nluc used in this study was about 25 times brighter than Rluc8, and the dynamic range calculated as a luminescence ratio of Luphin in the range of pH 7.0 to 8.5 was greater than that of pHlash, suggesting that Luphin is more appropriate for use in photosynthetic organisms. On another note, a disadvantage of sensor proteins utilizing Rluc or Nluc is that the luminescence emitting reaction is accompanied by O_2_ consumption and CO_2_ production. For example, under light conditions, increased O_2_ production from PSII may promote the luciferase reaction. Therefore, the luminescence intensity from Luphin that is expressed in *Synechocystis* may be enhanced. In addition, since photosynthetic and respiratory electron transfers involve O_2_ production and consumption, the electron transfer rate may be affected by the O_2_ consumption during the luciferase reaction.

Luphin can be localized to subcellular organelles by fusing the targeting signal sequence because it is a gene-encoded sensor protein. For example, the pH dynamics in the chloroplast stroma is a great interest in the research field of photosynthesis, and Luphin must be a good tool for this purpose. Indeed, there is a report that the luminescence from the luciferase introduced into the chloroplast was successfully observed with keeping the shape of a leaf by using EM-CCD (29). In addition, the internal pH change caused by photosynthetic reactions in chloroplasts can be measured simultaneously with excitation of photosystems by a red light with a wavelength longer than 600 nm, because the emission wavelength of Luphin is shorter than 600 nm. Accordingly, Luphin must be an excellent tool for *in situ* measurements of pH dynamics in photosynthetic organisms.

### Promotion and suppression mechanism of the electron transfer on the thylakoid membranes of *Synechocystis*

In this study, we succeeded in the real-time monitoring of changes in the estimated pH in the cytoplasm by using Luphin that was expressed in *Synechocystis* cells. The observed changes should be mainly caused by changes in photosynthetic electron transfer activities (Fig. 5). The process of photosynthetic electron transfer and proton transfer at the thylakoid membrane of *Synechocystis* is briefly described as follows. First, the electrons, produced by the water-splitting reaction on the PSII, are transferred to PQ, and PQ is then reduced to PQH_2_. One electron of PQH_2_ is transferred to plastocyanin (PC) when PQH_2_ is oxidized at the Q_o_-site on cytochrome *b*_*6*_*f* (Cyt *b*_*6*_*f*). Simultaneously, the other electron is transferred to the Q_i_-site on Cyt *b*_*6*_*f*. The electron is used for the rereduction of PQ at the Q_i_-site. This electron circulation between the PQ pool and Cyt *b*_*6*_*f* is called the Q cycle (30). The electron of PC is transferred to Fd *via* PSI and eventually produces NADPH from NADP^+^. Proton influx is coupled with the electron transfer at both PSII and Cyt *b*_*6*_*f* and then generates ΔpH across thylakoid membranes. In addition, unlike green algae and green plants, cyanobacteria possess respiratory chain complexes in the thylakoid membranes. Therefore, respiratory electron transfer mainly occurs on thylakoid membranes under dark conditions.

In a series of observed changes in the estimated pH in the cytoplasm of *Synechocystis* (Fig. 5*A*), the transient pH decrease during the irradiation of white light and the pH increase after turning off the light were unpredicted results. The observed changes were possibly due to the reduction of the PQ pool (Fig. 6*A*) and PQH_2_ oxidation at Cyt *b*_*6*_*f* (Fig. 6*B*), which are suggested by the inhibition experiments. Based on these observed changes, we here propose the mechanism of electron transfer promotion and suppression in the thylakoid membranes as follows.

**Figure 7:**
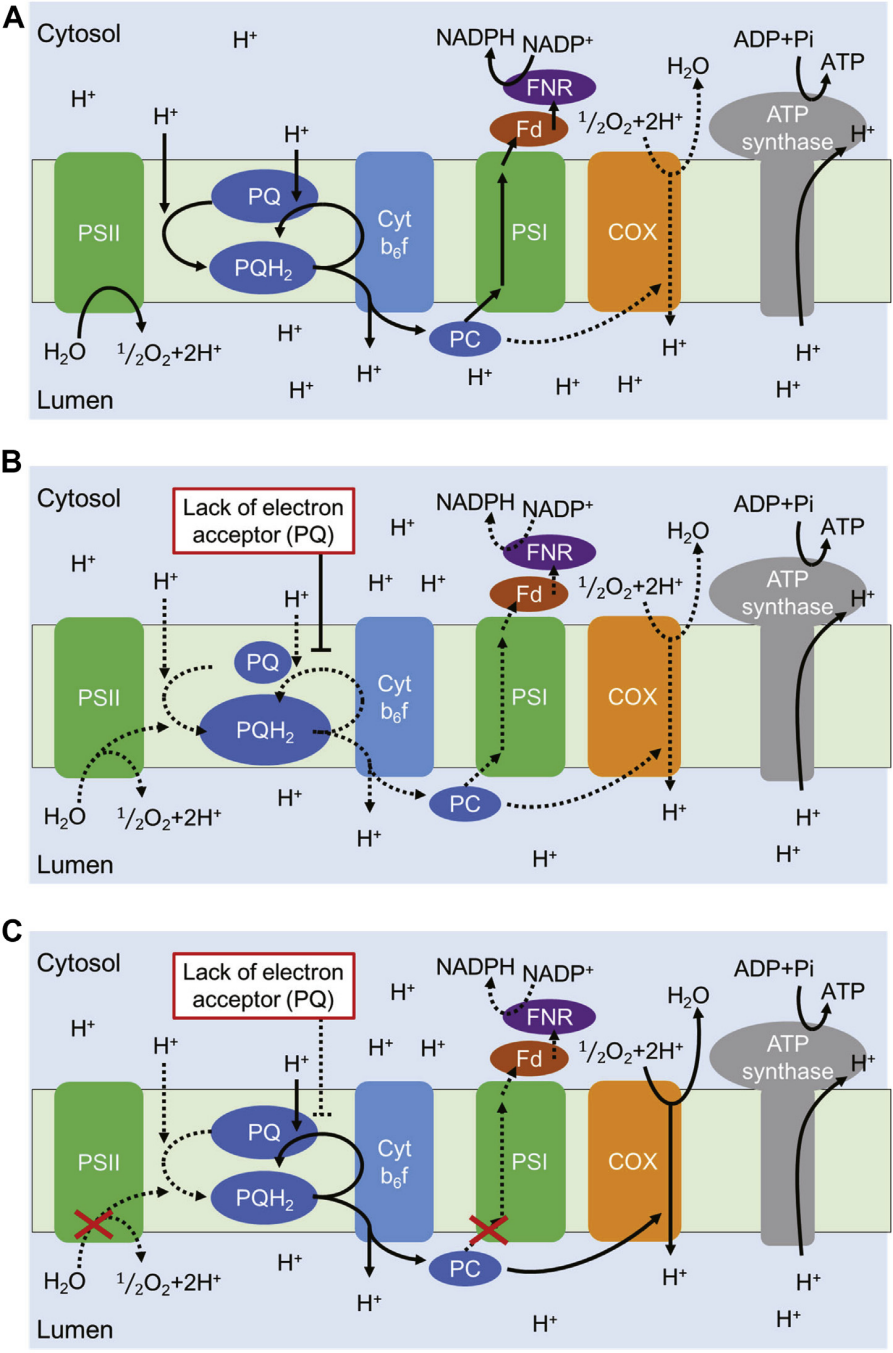
**The plausible model of the promotion and suppression of electron transfer in thylakoid membranes of *Synechocystis*.***A*, at 1 min after beginning the irradiation, photosynthetic electron transfer is promoted. *B*, a few minutes after the start of irradiation, plastoquinone (PQ), the electron acceptor for Cyt *b*_*6*_*f*, is deficient due to PQ pool reduction. This causes the suppression of photosynthetic electron transfer. *C*, at 1 min after turning the light off, PQ pool reduction at PSII stops. The PQ pool is then oxidized, and the shortage of PQ is relieved. This causes the promotion of electron transfer from the PQ pool to cytochrome c oxidase (COX) *via* Cyt *b*_*6*_*f*. The solid arrows indicate promoted electron and proton transfer pathways, and dashed arrows indicate suppressed electron and proton transfer pathways.

1. Within 1 min of irradiation, photosynthetic electron transfer is promoted and pH increase in the cytoplasm is induced (Fig. 7*A*).
2. The PQ pool is reduced a few minutes later, and PQ, which is required for electron transfer in the Q cycle, is deprived at the Q_i_-site on Cyt *b*_*6*_*f*. The photosynthetic electron transfer is then suppressed. Consequently, the formation rate of ΔpH becomes slower than that of ΔpH dissipation by ATP synthase, and therefore the pH decrease in the cytoplasm is initiated (Fig. 7*B*).
3. After turning off the light, PQ reduction at PSII is terminated and the PQ pool is rather oxidized. Therefore, the shortage of PQ at the Q_i_-site is relieved and the electron transfer at Cyt *b*_*6*_*f* is activated. Finally, the pH increase in the cytoplasm that accompanies PQH_2_ oxidation is observed, which is accumulated during irradiation. Under dark conditions, the electrons of PC must be consumed at the respiratory chain pathway, such as cytochrome c oxidase (COX) (Fig. 7*C*).

In chloroplast, the acidification of the lumen is thought to be one of the major factors regulating linear electron transport (see the review by Tikhonov (31)). It has also been reported that the thylakoid H^+^/K^+^ antiporter KEA3 in *Arabidopsis thaliana* contributes to the regulation of protonation of the thylakoid lumen (32). Because our sensor protein can visualize intracellular pH changes in real time (Fig. 5), we expect that appropriate introduction of the sensor protein into the mutant strains of these factors will allow us to evaluate the contribution of these factors to the proton transfer reaction and its regulation.

The results in Figure 5, *C*–*D* showed that the estimated pH increase coupled with photosynthetic electron transfer was small with higher light intensity. It has been reported that the photosynthetic parameter “qP” decreases with increasing light intensity, suggesting that the PQ pool is reduced with increasing light intensity (33). From this point of view, it is expected that photosynthetic electron transfer is suppressed by the reduced PQ pool, which consequently suppresses at Q_o_-site on Cyt *b*_*6*_*f*.

Taken together, Luphin enabled *in situ* pH measurements under fluctuating light conditions. By using this sensor protein, we can report the unknown electron transfer dynamics in thylakoid membranes.

## Experimental procedures

### Gene construction

To obtain EGFP_pH_, the gene for EGFP in the pET-23a vector (Novagen) was used as a template. The superfolder mutations and the other mutations were introduced into the desired genes using Overlap extension PCR (34) and PrimeSTAR Mutagenesis Basal Kit (Takara), respectively.

The cDNAs of EGFP_pH_ΔC12 and NlucΔN4 were amplified by PCR and fused with the intermediate random linker sequence of two amino acids using Hot Fusion methods (Fu *et al.* 2014). The linker sequence “W-T” was selected using an in-house random screening system.

The cDNA fragments encoding the mature protein-coding regions were cloned into pET-23a vector (Novagen) to express the protein with a C-terminal His-tag.

### Protein expression and purification

*Escherichia coli* (*E. coli*) strain BL21 (DE3) was transformed with each expression plasmid. Transformed *E. coli* was then cultured at 37 °C to reach OD_600_ = 0.2 ∼ 0.8. The expression of the desired protein was induced using 1 mM IPTG, followed by further cultivation at 21 °C overnight. The harvested cells were disrupted and ultracentrifuged (125,000*g* for 40 min) to obtain the supernatant containing the desired protein for subsequent purification. His-tagged proteins were purified *via* Ni–NTA affinity chromatography using the Ni–NTA agarose (QIAGEN) resin. The Ni–NTA agarose resin was washed in 25 mM Tris-HCl (pH 8.0) containing 20 mM imidazole and eluted using the same buffer containing 250 mM imidazole. The elusions were applied to a TOYOPEARL Butyl-650 column (TOSOH, Tokyo) and eluted using 20 mM Tris-HCl (pH 7.0) with an ammonium sulfate reverse gradient from 30% to 0%. The peak fractions eluted from the column were collected. The elution containing the desired proteins was dialyzed and concentrated with Amicon Ultra Filters (Merck Millipore).

### Fluorescence spectroscopy

Fluorescence excitation spectra were measured using an FP-8500 fluorescence spectrophotometer (JASCO, Tokyo). An emission intensity at 530 nm of EGFP_pH_ was measured at each excitation wavelength. The intensities were normalized at an excitation of 501 nm of pH 9.0 spectrum (Fig. 1*B*). An emission intensity by 480 nm excitation laser was also measured at each wavelength. The intensities were normalized at an emission of 513 nm of pH 9.0 spectrum (Fig. 1*C*). The sample solutions containing 1 μM EGFP_pH_ and 100 mM pH buffer (PIPES–NaOH for pH 6.0–7.0, HEPES–NaOH for pH 7.0–8.0, and Tricine–NaOH for pH 8.0–9.0) were used.

### Luminescence spectroscopy

Luminescence spectra were also measured using an FP-8500 fluorescence spectrophotometer. The excitation laser was shut off, and the emission intensity at each wavelength was measured. The sample solutions containing 2.5 nM Luphin, 0.2% Furimazine of Nano-Glo luciferase assay system (Promega), and 100 mM pH buffer (PIPES–NaOH for pH 6.0–7.2, HEPES–NaOH for pH 6.8–8.2, and Tricine–NaOH for pH 7.8–9.0) were used according to the instruction provided by the manufacturer.

### pH calibration

Luminescence intensities were measured using the multiplate reader, TriStar LB 941 (Berthold). To obtain the intensities in 420 to 460 nm and 495 to 540 nm, the luminescence was detected through a BA420-460 filter and a BA495-540GFP filter (Olympus), respectively. The exposure period was 0.5 s for each wavelength range. The contents of the sample solution were the same as those used in “Luminescence spectroscopy.” To confirm the physical stability of Luphin, NaCl, KCl, CaCl_2_, or MgCl_2_ was added.

### Culture of *Synechocystis*

*Synechocystis* and its Luphin expressing strain were cultured in BG-11 medium (35) buffered with 20 mM HEPES–KOH (pH 7.4). Cultures were grown at 30 °C under continuous light conditions (40 μmol photons m^−2^ s^−1^) and aerated with 1% (v/v) CO_2_.

### Construction of *Synechocystis* strain expressing Luphin

Luphin was expressed under the control of the PsbAⅡ promoter in *Synechocystis*. The cDNA of Luphin and the pTCP2031V vector were amplified by PCR using the following primer pairs: 5′-ATAAGGAATTATAACCATATGGCTAGCAAAGGAGA-3′ and 5′- ATCCAATGTGAGGTTAACTCAGTGGTGGTGATGGT-3′ for Luphin and 5′- CACCATCACCACCACTGAGTTAACCTCACATTGGA-3′ and 5′- TTCTCCTTTGCTAGCCATATGGTTATAATTCCTTA-3′ for pTCP2031V, respectively. The DNA fragments were fused using the Hot Fusion method. The pTCP2031V was originated from pTKP2031V (36, 37) and contained a chloramphenicol-resistant cassette instead of a kanamycin-resistant cassette. The resultant plasmid was transformed into *Synechocystis* by homologous recombination. Plasmids selected by DNA sequencing were mixed with *Synechocystis* cells, and the transformants were selected and subcultured in the presence of 20 μg ml^−1^ chloramphenicol.

### BRET measurement of *Synechocystis* strain expressing Luphin

The luminescence from *Synechocystis* expressing Luphin was measured using TriStar LB941, and the setting was the same as “pH calibration.” The measurement was conducted every 1 min. White light and FR light were applied at an interval time of 49 s, and their intensities were 40 μmol photons m^−2^ s^−1^. For FR light irradiation, an LED panel “ISLM-150 × 150 FF” (CSS) was used. The sample solution containing 90 μl bacterial solution and 10 μl Furimazine solution (1% Furimazine diluted by BG-11 medium) was used. To prepare the bacterial solution, the culture medium of OD_750_ = 0.2 to 0.4 was adjusted to OD_750_ = 0.2 by BG-11 medium and was adapted to the dark condition at room temperature for 30 min before mixing with Furimazine solution. Moreover, 10 min after mixing, the measurement was initiated. FCCP was stored at 3 mM in 50% (v/v) ethanol. DCMU and DBMIB were stored at 1 mM in 50% (v/v) ethanol.

### Photosynthetic activity measurements

Chlorophyll fluorescence was measured using Dual-PAM 100 (Walz, Germany). Prior to the measurements, *Synechocystis* cells were adjusted to OD_750_ = 0.5. The minimum chlorophyll fluorescence (Fo) was measured under dark conditions. Actinic light treatment (40 μmol photons m^−2^ s^−1^) was then delivered for 10 min to obtain the fluorescence parameters, steady-state fluorescence (F), and maximum fluorescence in the light (Fm’). After switching off the actinic light, 20 μM DCMU was added and maximum chlorophyll fluorescence (Fm) was determined after reirradiation of the actinic light. The photosynthetic parameters were calculated using the following equations;

Fv/Fm=(Fm−Fo)/Fm

ΦII=(Fm′−F)/Fm′

NPQ=(Fm/Fm′)/Fm′

as described (38).

## Data availability

All data are contained within the article and can be shared upon request (thisabor@res.titech.ac.jp).

## Conflict of interest

The authors declare that they have no conflicts of interest with the contents of this article.
