# Supplementary material for: A luminescent Nanoluc-GFP fusion protein enables readout of cellular pH in photosynthetic organisms
Source: J Biol Chem. 2020 Dec 4;296:100134. doi: 10.1074/jbc.RA120.016847 (PMC7948502; doi:10.1074/jbc.RA120.016847)
Supplement: Supplementary Figures and Tables [file mmc1.pdf]

Supplementary Fig.1. The absorption spectra of Luphin in buffers with various pH values. Luphin (10 μM) was used for the measurement.

Supplementary Fig. 2. Expression of Luphin in *Synechocystis* cell. Luphin expressed in the cyanobacteria was detected by the Western blotting method using the GFP antibody. The total proteins extracted from the cells were loaded as indicated (1, 5, 10 μg). Rec, 50 ng of recombinant Luphin.

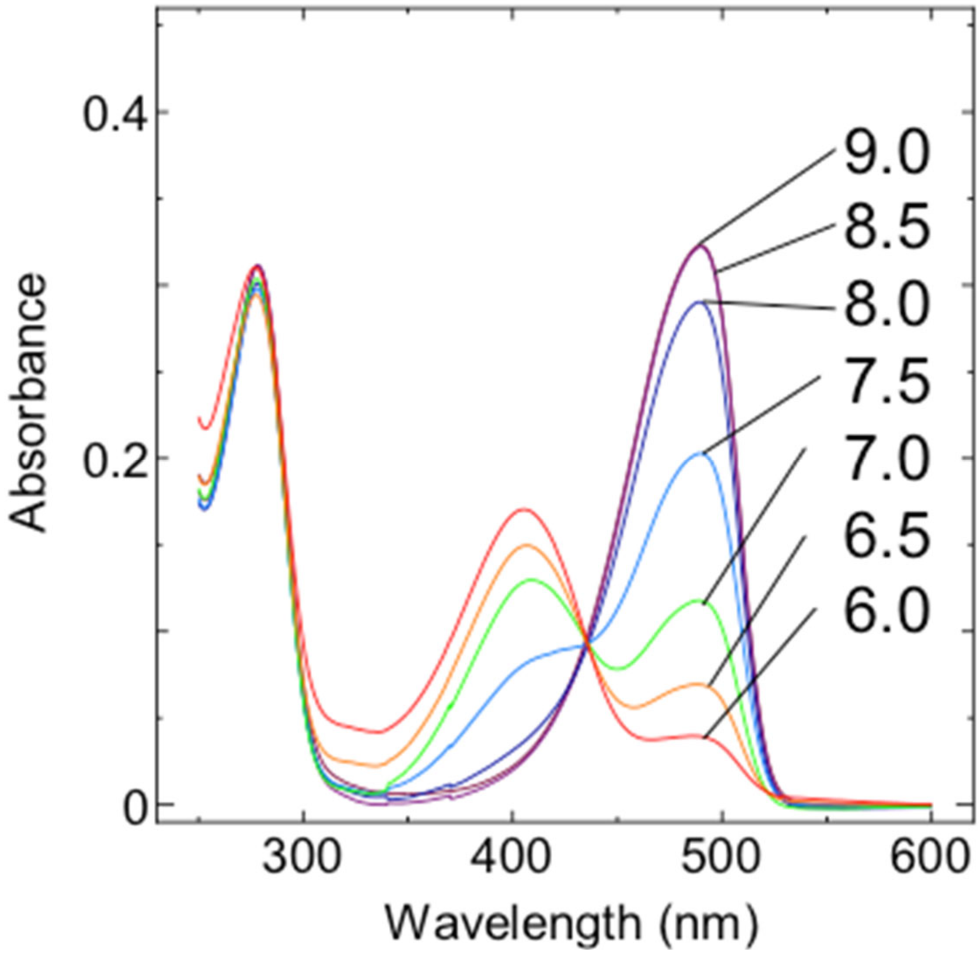

Supplementary Fig. S1

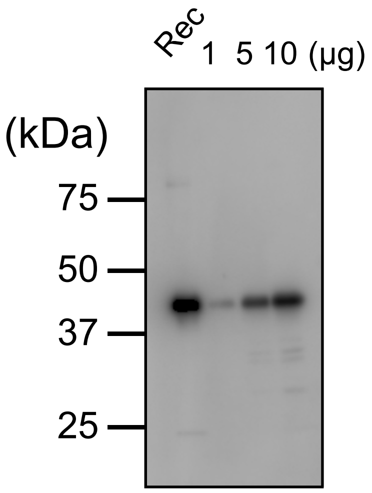

Supplementary Fig. S2

Supplemental Table S1. Photosynthetic parameters of a Luphin-expressing (LUPHIN) and the wild-type (WT) *Synechocystis* cells

	LUPHIN + Fur	LUPHIN + BG11	WT + Fur	WT + BG11
Fv/Fm	0.4536 ± 0.0046	0.4611 ± 0.0028	0.4605 ± 0.0035	0.4609 ± 0.0101
ΦII	0.2954 ± 0.0098	0.2981 ± 0.0060	0.2943 ± 0.0089	0.2980 ± 0.0082

The means ± SD from three independent experiments are shown.
*ΦII values were determined from the values at 600 seconds after actinic light (red, 40 µmol m^{-2} s^{-1}) exposure.
